# A 3D Printed Modular Soft Gripper Integrated With Metamaterials for Conformal Grasping

**DOI:** 10.3389/frobt.2021.799230

**Published:** 2022-01-07

**Authors:** Charbel Tawk, Rahim Mutlu, Gursel Alici

**Affiliations:** ^1^ School of Mechanical, Materials, Mechatronic and Biomedical Engineering and Applied Mechatronics and Biomedical Engineering Research (AMBER) Group, University of Wollongong, Wollongong, NSW, Australia; ^2^ ARC Centre of Excellence for Electromaterials Science, University of Wollongong Innovation Campus, North Wollongong, NSW, Australia; ^3^ Faculty of Engineering and Information Sciences, Dubai Knowledge Park, University of Wollongong in Dubai, Dubai Knowledge Park, Dubai, United Arab Emirates

**Keywords:** grasping, modular robot, soft gripper, soft robot, soft pneumatic actuator, metamaterial, 3D printing

## Abstract

A single universal robotic gripper with the capacity to fulfill a wide variety of gripping and grasping tasks has always been desirable. A three-dimensional (3D) printed modular soft gripper with highly conformal soft fingers that are composed of positive pressure soft pneumatic actuators along with a mechanical metamaterial was developed. The fingers of the soft gripper along with the mechanical metamaterial, which integrates a soft auxetic structure and compliant ribs, was 3D printed in a single step, without requiring support material and postprocessing, using a low-cost and open-source fused deposition modeling (FDM) 3D printer that employs a commercially available thermoplastic poly (urethane) (TPU). The soft fingers of the gripper were optimized using finite element modeling (FEM). The FE simulations accurately predicted the behavior and performance of the fingers in terms of deformation and tip force. Also, FEM was used to predict the contact behavior of the mechanical metamaterial to prove that it highly decreases the contact pressure by increasing the contact area between the soft fingers and the grasped objects and thus proving its effectiveness in enhancing the grasping performance of the gripper. The contact pressure can be decreased by up to 8.5 times with the implementation of the mechanical metamaterial. The configuration of the highly conformal gripper can be easily modulated by changing the number of fingers attached to its base to tailor it for specific manipulation tasks. Two-dimensional (2D) and 3D grasping experiments were conducted to assess the grasping performance of the soft modular gripper and to prove that the inclusion of the metamaterial increases its conformability and reduces the out-of-plane deformations of the soft monolithic fingers upon grasping different objects and consequently, resulting in the gripper in three different configurations including two, three and four-finger configurations successfully grasping a wide variety of objects.

## 1 Introduction

Low elastic moduli materials and smart structures that are inspired by nature empower soft robots to perform tasks by mechanically adapting their bodies to dynamic environments by undergoing extremely large deformations without any sign of material or structural failures due to their inherent softness. Soft-bodied species inspiring soft roboticists include but are not limited to elephant trunks, octopus arms, worms, and caterpillars (Trivedi et al., 2008; Huai-Ti et al., 2011). Soft robots are characterized by their adaptability, conformability, agility, and durability (Whitesides, 2018) compared to their conventional rigid and stiff counterparts (Alici, 2018). Soft robotic concepts can be used in a wide variety of applications such as soft grippers (Shintake et al., 2018; Zhou et al., 2021), locomotion robots (Calisti et al., 2017), medical devices (Cianchetti et al., 2018), and human-machine interfaces (Tawk and Alici, 2021).

Conventional robotic grippers have been extensively studied for repetitive tasks involving picking and placing a variety of objects with different weights, shapes, sizes, textures, and stiffnesses. However, traditional grippers are made of stiff materials and rigid components that make them unsuitable to operate safely alongside humans and in unstructured and dynamic environments. The fabrication of traditional grippers requires complex machining and laborious assembly processes. Also, multiple sensors are required along with complex control algorithms, to ensure that a sufficient but not excessive grasping force is applied without damaging the objects being handled (Pham and Yeo, 1991). This being said, grasping delicate objects in dynamic environments using conventional grippers requires complex control methods with reliable sensory feedback to minimize the possibility of damaging the objects being handled.

Soft grippers that are made of highly deformable and compliant materials and structures are perfect candidates for handling (Shintake et al., 2018) and manipulating (Abondance et al., 2020) delicate objects. First, these soft grippers can be fabricated using low-cost and commercially available soft materials (Tawk et al., 2018). Second, they can handle a wide variety of objects with different stiffnesses without requiring any sensory feedback and control systems since contact forces are highly reduced (Tawk et al., 2019a). Finally, due to their inherent softness, they are safe to operate alongside humans and in dynamic environments. The development of universal grippers that can pick arbitrary objects remains a challenge for soft and rigid grippers. To achieve a stable grip, in both static and dynamic conditions, a large contact area between the object being handled and the gripper is required.

A soft robotic gripper can generate highly passive deformations and adapt itself to the shape of an object being handled due to its inherent compliance which is a characteristic of soft robotic systems (Laschi and Cianchetti, 2014). Many of the soft grippers are actuated using positive pressure, negative (i.e., vacuum) pressure, or a combination of positive and negative pressure (Fatahillah et al., 2020) soft pneumatic actuators. Based on soft pneumatic actuators and three-dimensional (3D) printing different soft grippers with complex topologies can be developed to generate various modes of deformation. The main soft pneumatic actuators used in soft grippers are PneuNets (Mosadegh et al., 2014; Hao et al., 2016; Alici et al., 2017; Glick et al., 2018; Wang et al., 2018; Hu and Alici, 2020) and fiber-reinforced actuators (Deimel and Brock, 2016; Fraś et al., 2017; Zhou et al., 2017; Zhang et al., 2018) where the actuators are fabricated using additive manufacturing techniques such as fused deposition modeling (FDM) (Yap et al., 2016; Mutlu et al., 2017; Keong and Hua, 2018; Tawk et al., 2018; Tawk et al., 2019b; Tawk et al., 2019c), multi-material three-dimensional (3D) printing (MacCurdy et al., 2016) and silicone 3D printing (Schaffner et al., 2018; Yirmibeşoğlu et al., 2019), or conventional soft robotic manufacturing techniques that require complicated and laborious fabrication steps (Marchese et al., 2015).

Also, the versatility and enhanced conformability of soft robotic grippers are achieved using various mechanical designs and bioinspired structures and such as kirigami shells (Yang et al., 2021), origami structures (Li et al., 2019), cellular structures (Kaur and Kim, 2019), bioinspired spiral springs (Zolfagharian et al., 2021), bionic torus (Zang et al., 2020), torus inspired mechanism (Sui et al., 2020), suction cups with elastomer films (Koivikko et al., 2021), cylindrical accordion structures with gecko-like skins (Hao et al., 2021), compliant mechanisms and fingers (Chen et al., 2018; Hussain et al., 2020), reconfigurable fingers (Pagoli et al., 2021), monolithic underactuated fingers (Mutlu et al., 2016), and a combination of 3D printed suction cups and complaint soft fingers (Tawk et al., 2019b).

**FIGURE 1:**
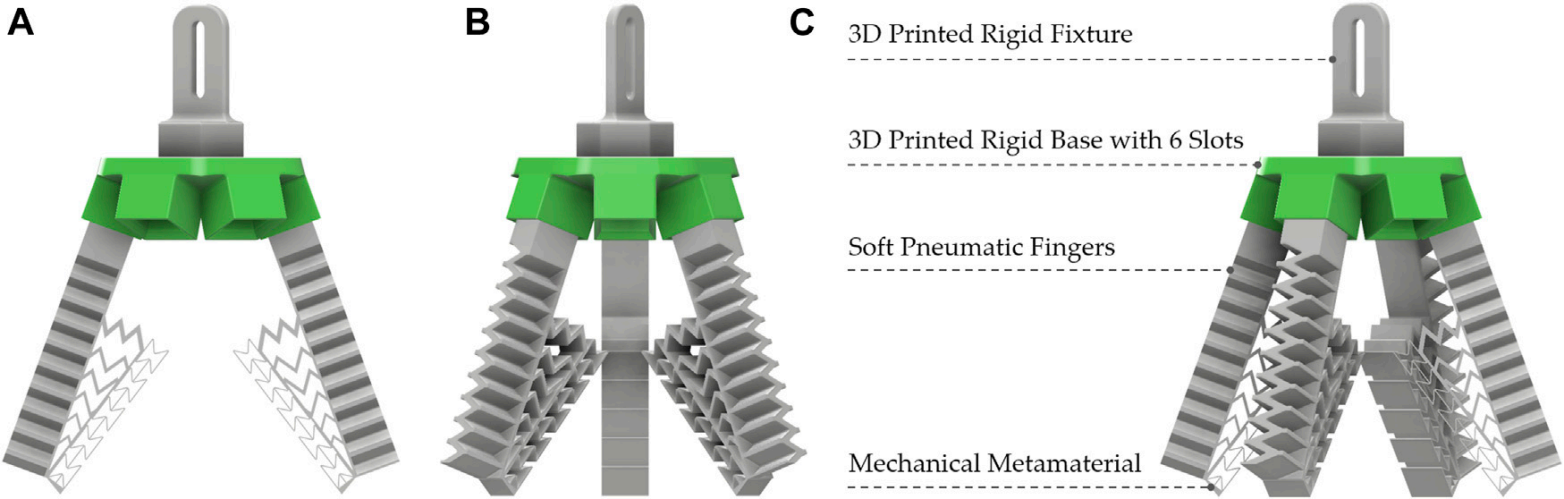
The main components of the 3D printed modular soft gripper with integrated metamaterial. **(A)** Two-finger configuration. **(B)** Three-finger configuration. **(C)** Four-finger configuration.

In this work, we present 3D printed modular soft pneumatic gripper with integrated mechanical metamaterial for conformal grasping (Figure 1) which was 3D printed from a commercially available thermoplastic poly (urethane) (TPU) (Tawk et al., 2020). The monolithic pneumatic fingers of the gripper along with the mechanical metamaterial were 3D printed without requiring any support material and postprocessing in a single manufacturing step. Each soft pneumatic finger has a soft mechanical metamaterial that is composed of an auxetic structure along with compliant ribs as shown in Figure 1.

The integrated mechanical metamaterial consisting of an auxetic structure and compliant ribs increased dramatically the conformability of the fingers (i.e., the gripper) by increasing the contact area and reducing the contact pressure as demonstrated by finite element modeling (FEM). FEM was also employed to predict the behavior of the soft fingers and optimize their performance in terms of deformation and tip force. In addition, the experimental deformation (i.e., bending angle) and tip force of the soft fingers were characterized and compared to their FEM counterparts to show the accuracy of the numerical models considered. The configuration of the gripper can be easily and rapidly modulated by changing the number of soft fingers used to meet certain manipulation requirements or constraints.

To this aim, two-dimensional (2D) and 3D dimensional grasping performance of three different configurations (i.e., two-finger, three-finger, and four-finger configuration) were assessed using a series of grasping experiments. The integration of the mechanical metamaterial allows the gripper to grasp different objects successfully compared to the same configurations where the mechanical material is not included which resulted in the gripper failing to grasp any of the same objects.

The inclusion of the mechanical metamaterial played a significant role not only in successfully grasping the objects or stably gripping them, but also proved that the added capability of conformability leads to reduced out-of-plane deformations that also increased the gripping stability. Thus, the grasping performance of the soft modular gripper with the integrated metamaterial was considerably enhanced as the gripper could successfully grasp different objects. The soft modular gripper is a great candidate for universal grasping and handling a variety of fruits and vegetables.

The contributions of this paper are to 1) offer soft monolithic soft pneumatic actuators (i.e., fingers) with integrated metamaterial that can be easily and directly manufactured in a single step using a low-cost and open-source FDM 3D printer, 2) characterize the soft monolithic fingers experimentally and predict their deformation behavior, tip force and contact behavior accurately using FEM to quickly and efficiently design their structure, and 3) implement such soft monolithic fingers in a modular gripper to prove through its 2D and 3D grasping performance characterization that such fingers with integrated metamaterial enhance the grasping performance of the soft modular gripper by increasing its conformability and reducing its fingers’ out-of-plane deformations.

## 2 Materials and Methods

### 2.1 Modeling and Fabrication

**FIGURE 2:**
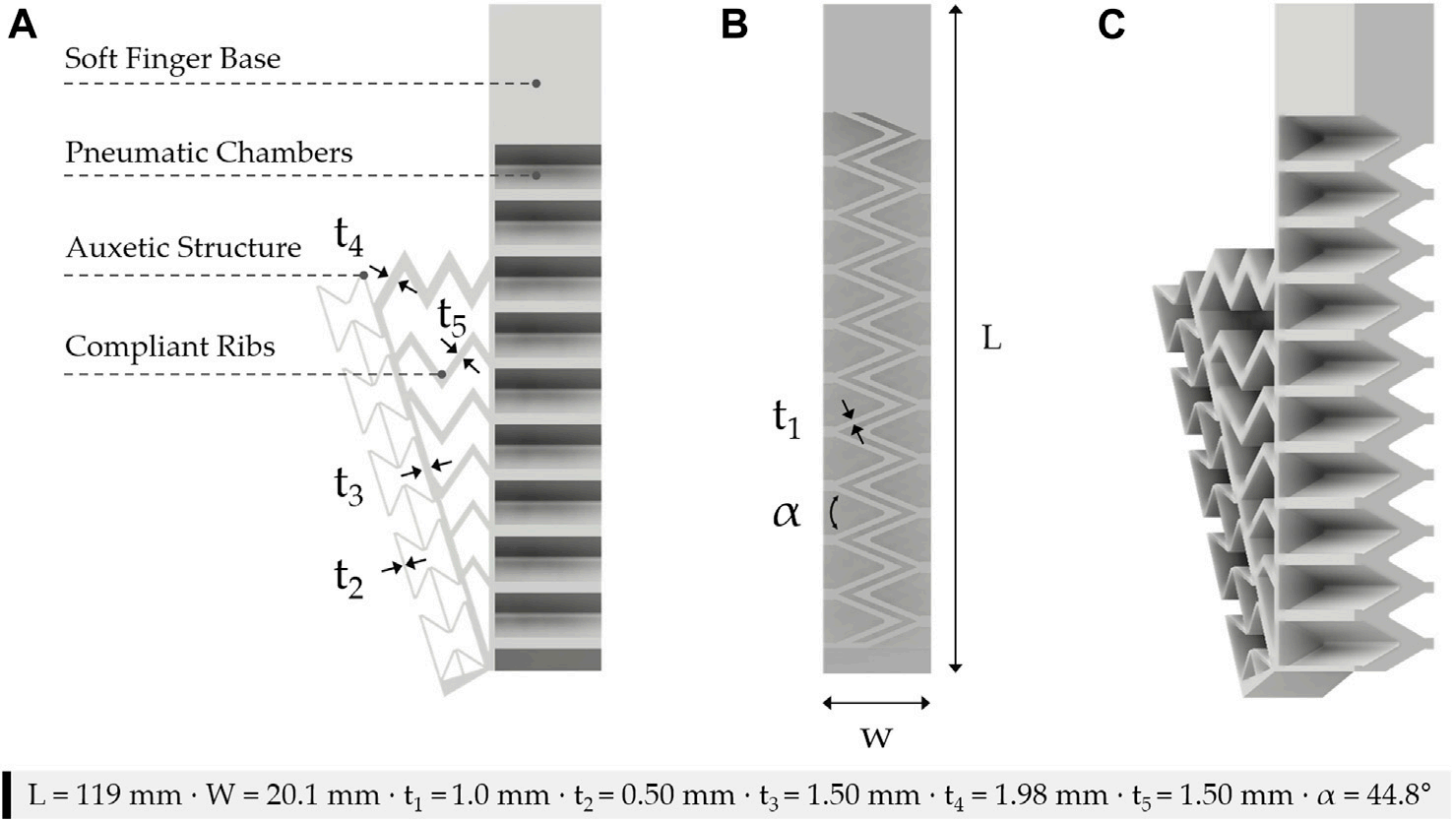
3D printed soft monolithic finger with mechanical metamaterial **(A)** three-dimensional view, **(B)** top view, and **(C)** side view.

A low-cost and open-source FDM 3D printer (FlashForge Inventor, FlashForge Corporation) along with a TPU that is known commercially as NinjaFlex (NinjaTek, United States) were used to fabricate the monolithic soft pneumatic fingers along with the mechanical metamaterial of the soft gripper. Autodesk Fusion 360 (Autodesk Inc.) was used to design the computer-aided-design (CAD) models of the soft gripper. A commercially available slicer (Simplify3D LLC, OH) was used to slice the CAD models where the 3D printing parameters listed in Table 1 were optimized to obtain airtight soft pneumatic structures (Tawk et al., 2018). The soft fingers of the gripper were printed along their width (W, Figure 2) to ensure that no support material is required during the 3D printing process.

**TABLE 1:** Optimized parameters in Simplify3D (Version 4.1.2) for 3D printing airtight soft monolithic pneumatic actuators with integrated metamaterial.

Parameter	Value	Unit
Resolution settings		
Primary Layer Height	0.1	mm
First Layer Height	0.09	mm
First Layer Width	0.125	mm
Extrusion Width	0.4	mm
Ooze Control		
Coast at End	0.2	mm
Retraction Settings		
Retraction Length	4	mm
Retraction Speed	40	mm/s
Speed Settings		
Default Printing Speed	10	mm/s
Outline Printing Speed	8	mm/s
Solid Infill Speed	8	mm/s
First Layer Speed	8	mm/s
*X*/*Y* Axis Movement Speed	50	mm/s
*Z* Axis Movement Speed	20	mm/s
Temperature Settings		
Printing Temperature	240	°C
Heat Bed Temperature	32	°C
Cooling Settings		
Fan Speed	50	%
Infill Settings		
Infill Percentage	100	%
Infill/Perimeter Overlap	30	%
Thin Walls		
Allowed Perimeter Overlap	15	%
External Thin Wall Type	Perimeters Only	—
Internal Thin Wall Type	Allow Single Extrusion Fill	—
Movement Behavior		
Avoid Crossing Outline	ENABLED	—
Allowed Detour Factor	100	—
Additional Settings		
Extrusion Multiplier	1.15	—
Top Solid Layers	10	—
Bottom Solid Layers	10	—
Outline/Perimeter Shells	4	—
Support Material Generation		
Support Type	DISABLED	—

### 2.2 Modular Soft Gripper Design

The soft pneumatic finger with the mechanical metamaterial is shown in Figure 2. The dimensions of the soft pneumatic finger and the metamaterial shown in Figure 2 were chosen based on the FEM studies for assessing the behavior of the soft finger and the metamaterial and to ensure that the 3D printed soft pneumatic fingers are airtight. A series of designs were considered for the soft mechanical metamaterial and simulated to enhance its deformation and behavior and to ensure that the fingers of the modular gripper can achieve conformal grasping with a wide variety of shapes. The soft fingers were designed to generate the bending motion required and to deliver the grip forces required for grasping numerous objects. A Zig-Zag structure was chosen in the design process of the soft fingers to eliminate any contact between the walls of the adjacent chambers upon actuation. This Zig-Zag design prevents any energy losses due to the contact between adjacent pneumatic chambers as in conventional PneuNets soft actuators.

The bottom layer of the fingers acts as a strain-limiting layer that prevents them from extending along their length (L, Figure 2). The soft pneumatic fingers are the active component of the gripper whereas the soft mechanical metamaterial is the passive component. The dimensions of a single soft monolithic finger along with the dimensions of its mechanical metamaterial are shown in Figure 2.

### 2.3 Finite Element Modeling

**FIGURE 3:**
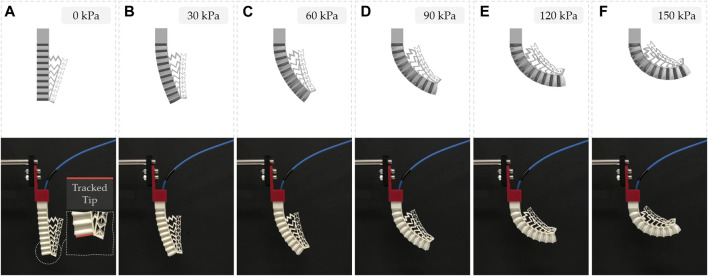
3D printed soft monolithic finger FEM **(top)** and experimental **(bottom)** bending behavior under different applied positive pressures. Inset: Tracked line for bending angle measurement using the vision processing algorithm.

**FIGURE 4:**
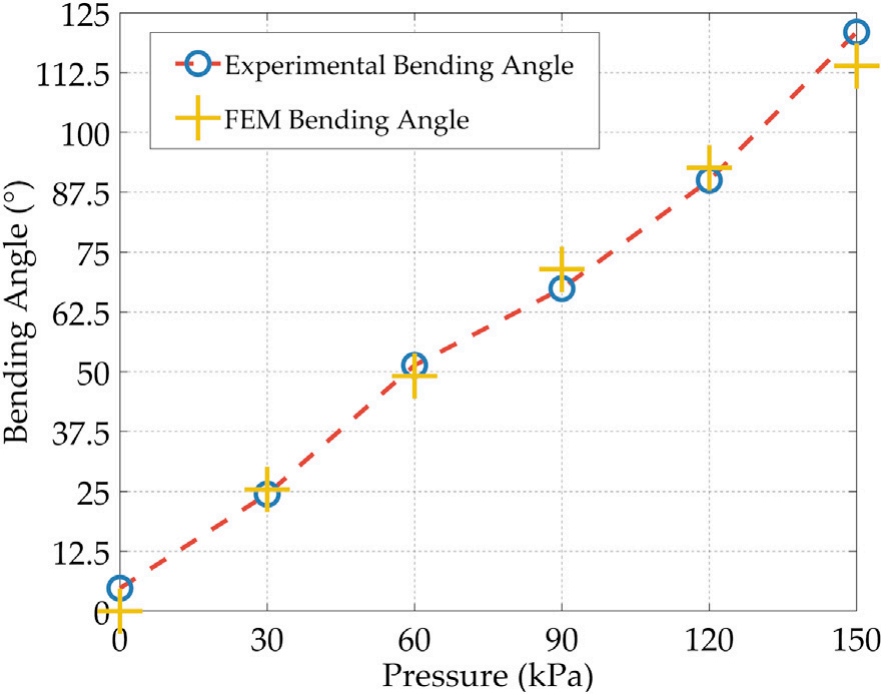
Experimental and FEM bending angle for the soft monolithic finger under different applied positive pressures.

**FIGURE 5:**
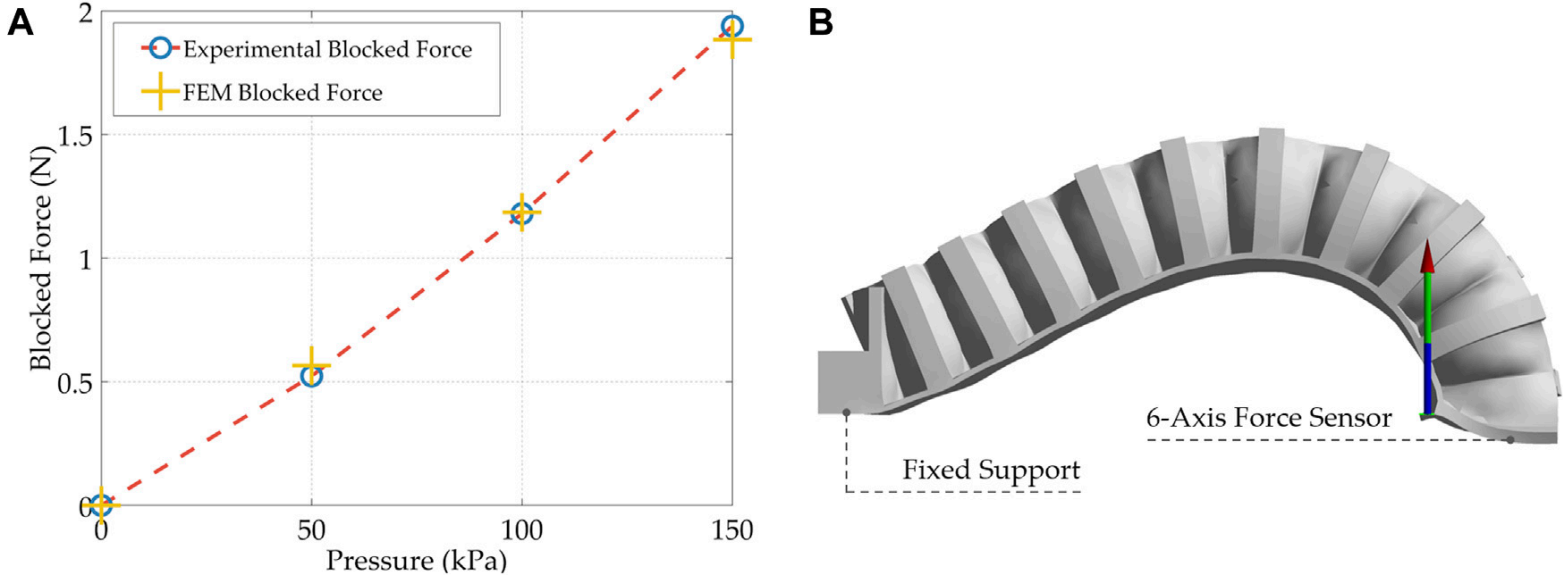
Tip force results and setup. **(A)** Experimental and FEM tip forces for the soft monolithic finger under different applied positive pressures. **(B)** Tip force measurement setup.

FE simulations were performed on a soft pneumatic finger to predict its behavior and optimize its topology based on the dimensions stated in Figure 2 to achieve the final design. A 5-parameter Mooney-Rivlin hyperelastic material model was developed based on the TPU experimental stress-strain data for use in ANSYS Workbench (ANSYS Inc.) (Tawk et al., 2018). The 3D CAD models of the fingers were meshed using higher-order tetrahedral elements where the mesh was studied to verify that the results are mesh independent. A fixed support boundary condition was applied at the base of the finger to fix it and a normal pressure was applied at its internal walls. In addition, contact pairs were defined between adjacent walls in the mechanical metamaterial that come into contact upon deformation. The objects were modeled using Structural Steel material available in ANSY due to its high stiffness since the objective was to assess the deformation behavior of the soft deformable metamaterial. Also, a frictional contact pair was defined between the objects and the soft fingers. The bending behavior (i.e., bending angle) of the actuator and its tip force were accurately predicted in the FE simulations. The bending behavior of the soft monolithic finger at different input pressures is shown in Figure 3. In addition, the FEM bending angles and the FEM tip forces are shown in Figure 4 and Figure 5, respectively.

**FIGURE 6:**
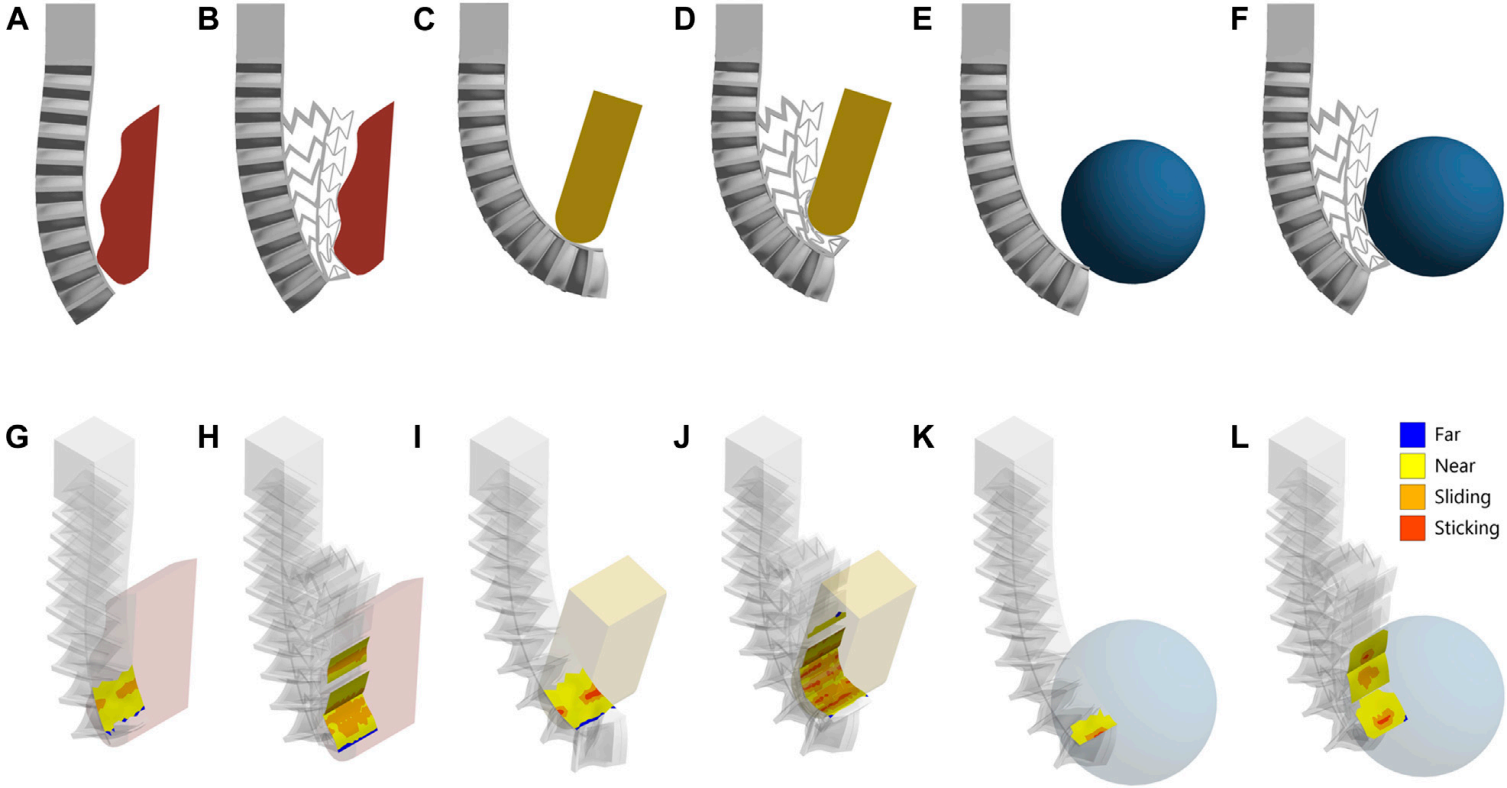
3D printed soft monolithic finger FEM contact simulations. The soft finger without and with the mechanical metamaterial in contact with **(A)** and **(B)** an irregular shape, **(C)** and **(D)** a bar with a rounded tip, and **(E,F)** a sphere. **(G–L)** The soft finger, without and with the mechanical metamaterial, contact status in each of the corresponding cases. For the contact legend, “Far” means there is no contact at all between the surfaces, “Near” means that the surfaces have normal separation within a pinball radius, “Sliding” means that the surfaces can slide relative to each other, and “Sticking” means that there is no movement between the surfaces in contact.

Also, the FE simulations were performed to assess the performance and predict the behavior of the mechanical metamaterial when it comes into contact with different shapes upon activation of a single finger with positive pressure as shown in Figure 6. For each object, the simulation was performed without including the mechanical metamaterial and with the inclusion of the mechanical metamaterial to show the difference in the behavior of the soft monolithic fingers and the difference in the contact pressure and area. The FEM proves that the contact area increases with the inclusion of the mechanical metamaterial which adapts to the surface of the objects in contact as shown in Figure 6. Consequently, the contact pressure dramatically decreased by up to 8.5 times as presented in Table 2.

**TABLE 2:** Contact pressure between the soft finger and the grasped shapes without and with the integrated metamaterial.

Shape	Contact Pressure CP1 (MPa)	Contact Pressure CP2 (MPa)	Ratio (CP1/CP2)
Integrated Metamaterial	No (Without Metamaterial)	Yes (With Metamaterial)	
Irregular	0.098	0.023	4.276
Sphere	0.565	0.113	5.011
Rounded Bar	0.262	0.031	8.574

It is important to note the three cases assessed using FEM to show conformability were chosen since they align with the application of the soft modular gripper in terms of grasping different objects. These cases do not represent all possible grasping cases and possibilities. However, in these cases, the objects, and their respective positions are well aligned with the experimental grasping tests conducted using the soft modular grippers.

With the inclusion of the mechanical metamaterial, the contact area between the actuator and the objects increases as shown in Figure 6. It is also verified experimentally in Section 3 that the soft gripper in its three different configurations cannot achieve a stable grasp or successfully grasp the different objects when the mechanical metamaterial is not included in its monolithic fingers. Both the FEM and experimental results proved that the mechanical metamaterial included in each finger of the gripper is necessary to achieve conformability by dramatically increasing the contact area and consequently reducing the contact pressure and reducing out-of-plane deformations.

It was proved that conformability improves the payload of soft grippers and their grasping capability as demonstrated by using fiber-reinforced actuators with conformal sleeves (Galloway et al., 2013). In addition, soft grippers conform to an object by making a contact along a surface to match the shape of the object being handled and therefore enhance their corresponding “shape matching” capability (Zhou et al., 2015). This finite contact (i.e., surface contact) was achieved by implementing the mechanical metamaterial to improve the conformability of the bending fingers. As demonstrated in the FEM and experimental results, before adding the mechanical metamaterial, the fingers established a point contact or line contact with the objects being handled. However, after adding the mechanical metamaterial, a surface contact that matches the shape of the objects being grasped was achieved and therefore, enhancing the conformability (i.e., shape matching) of the gripper. In addition, conformability simplifies the complexity of actuation, manipulation, control, and sensing by leveraging the inherent physical intelligence of soft robotic systems including soft grippers (Ke et al., 2021).

## 3 Results and Discussion

### 3.1 Bending Deformation and Tip Force Characterization

#### 3.1.1 Bending Deformation

The experimental bending deformation of the soft gripper was measured in MATLAB (The MathWorks Inc.) using a vision processing algorithm that tracked the bending angle of the soft finger at its tip under different applied positive pressures (Figure 3). The same bending angles were directly measured in the FEM simulations (Figure 3). The experimental and FEM bending angles at different applied pressures are shown in Figure 4 and Supplementary Video S1.

The FEM simulations predicted the experimental bending angles with great accuracy with a maximum difference of 5.999% at 150 kPa and a minimum difference of 2.8911% at 120 kPa. It is important to note that when no pressure was applied the experimental bending angle was not exactly 0° as expected, instead 4.76°, since the actuator is not capable of recovering fully its initial shape (Supplementary Video S1). This is mainly due to the properties of the soft TPU used. Although the TPU is soft and flexible, it does not fully recover its original shape such as silicones upon the removal of an applied mechanical deformation showing viscoelastic properties that are neglected in the material model used in the FE simulations.

#### 3.1.2 Tip Force

A 6-axis force sensor (K6D27, ME-Meßsysteme GmbH) was used to measure the tip force of a single finger. The finger was fixed at one end where the input pressure tube is located, and its tip was laid on the center of the force sensor. The pressure was ramped up by a step of 50 kPa to reach a maximum safe operating pressure of 150 kPa when the force was recorded. This value of 150 kPa was chosen to ensure that safety requirements were met even though the finger is capable of generating higher forces at higher input pressures. The experimental tip force at 150 kPa generated by a single cantilevered finger is 1.94 N. Again, the FEM simulations predicted the experimental tip force with great accuracy with a maximum difference of 8.0329% at 50 kPa and a minimum difference of 0.4153% at 100 kPa.

The FEM simulations predicted both the experimental bending deformation and tip force of the soft monolithic finger with great accuracy proving that FEM can be used in the design process of 3D printable soft actuators to optimize and predict their performance accurately before their fabrication (Tawk and Alici, 2020; Xavier et al., 2021). This approach makes the design process very efficient by saving huge amounts of time and potential fabrication resources.

### 3.2 Grasping Performance Characterization

The soft gripper can grasp a variety of objects. In this section, the soft gripper 2D and 3D grasping performance are evaluated using three different configurations including two, three, and four-finger configurations, with and without the inclusion of the mechanical metamaterial, to prove that the inclusion of the metamaterial makes the soft gripper conformal and able to grasp different objects successfully.

#### 3.2.1 2D Grasping Performance

**FIGURE 7:**
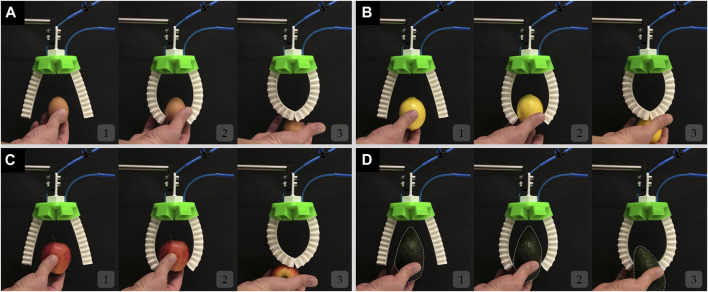
The soft modular gripper in a two-finger configuration without the mechanical metamaterial attempts to but fails to grasp **(A)** an egg, **(B)** a lemon, **(C)** an apple, and **(D)** an avocado.

**FIGURE 8:**
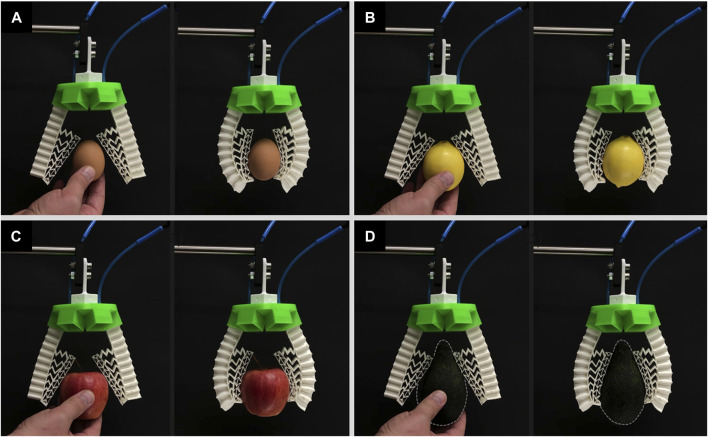
The soft modular gripper in a two-finger configuration with the mechanical metamaterial grasping successfully **(A)** an egg, **(B)** a lemon, **(C)** an apple, and **(D)** an avocado.

Figure 7 and Supplementary Video S2 show a two-finger configuration, where the fingers are not equipped with the mechanical metamaterial. As shown the gripper is not capable of holding the objects grasped including an egg, a lemon, an apple, and an avocado. Although the fingers of the gripper are soft, they cannot adapt to the shape of the objects being grasped. The fingers curl as expected for such soft pneumatic actuators, and only their tips come into contact with the objects being grasped. Similar behavior was also observed in the FEM simulations (Figure 6). Such behavior limits the contact area between the fingers of the gripper and the objects being handled which in turn limits the grasping capabilities of the gripper. However, a two-finger configuration gripper with the inclusion of the mechanical metamaterial can successfully hold the same objects being grasped as shown in Figure 8 and Supplementary Video S2. This result proves that the addition of the mechanical metamaterial which makes the soft monolithic fingers of the gripper conformal is necessary to achieve successful 2D grasps.

#### 3.2.2 3D Grasping Performance

The 3D printed rigid circular green base of the modular gripper contains six slots that are equally distributed where the number of pneumatic soft fingers can be modulated. For the 3D grasping performance evaluation, a three-finger configuration and a four-finger configuration are considered to grasp the same objects including an egg, a lemon, an apple, and an avocado.

**FIGURE 9:**
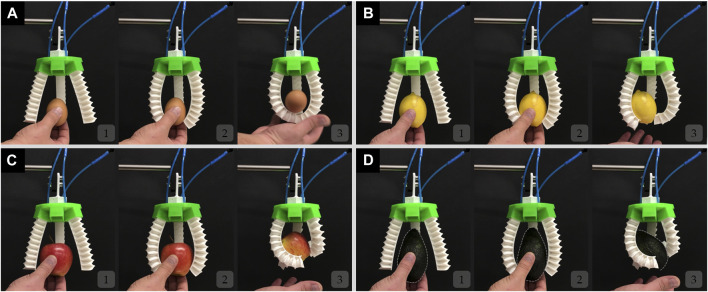
The soft modular gripper in a three-finger configuration without the mechanical metamaterial attempts to but fails to grasp **(A)** an egg, **(B)** and fails to grasp stably a lemon, **(C)** an apple, and **(D)** an avocado.

For the three-finger configuration modular gripper without the inclusion of the mechanical metamaterial, again the gripper was not capable of grasping the egg as shown in Figure 9 and Supplementary Video S3. For the lemon, apple, and avocado, although the soft gripper without the mechanical metamaterial could grasp the objects its fingers curled leading to out-of-plane deformations which in turn led to an unstable grip (Figure 9 and Supplementary Video S3). This curling behavior may be due to multiple factors including unequal gripping angles or forces (i.e., identical contact on each fingertip), and slightly different lengths of insertion for the fingers in the slots of the base resulting in length difference among the fingers.

**FIGURE 10:**
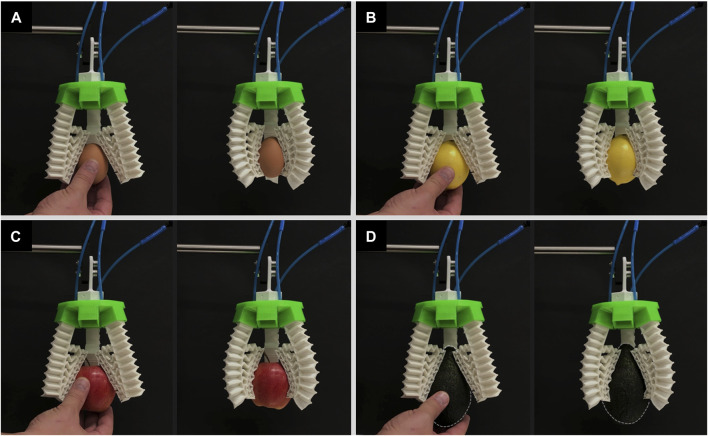
The soft modular gripper in a three-finger configuration with the mechanical metamaterial grasping successfully **(A)** an egg, **(B)** a lemon, **(C)** an apple, and **(D)** an avocado.

The same three-finger modular gripper with the inclusion of the mechanical metamaterial was able to successfully grasp all the objects by conforming to their shape and it alleviated the out-of-plane deformation behavior encountered and thus led to a stable and firm grasp as shown in Figure 10 and Supplementary Video S2. Again, the mechanical metamaterial proved that its addition is necessary not only to achieve conformability but to enhance the stability of the grip by highly reducing the out-of-plane deformation of each of the soft monolithic actuators. It is proved that out-of-plane deformations in soft actuators including twisting and sidewards bending have a negative effect on the grasping stability whenever such actuators are used for soft gripping (Scharff et al., 2019). Thus, reducing out-of-plane deformations lead to better grasping stability in soft grippers that are based on positive pressure soft pneumatic bending actuators (Scharff et al., 2019).

**FIGURE 11:**
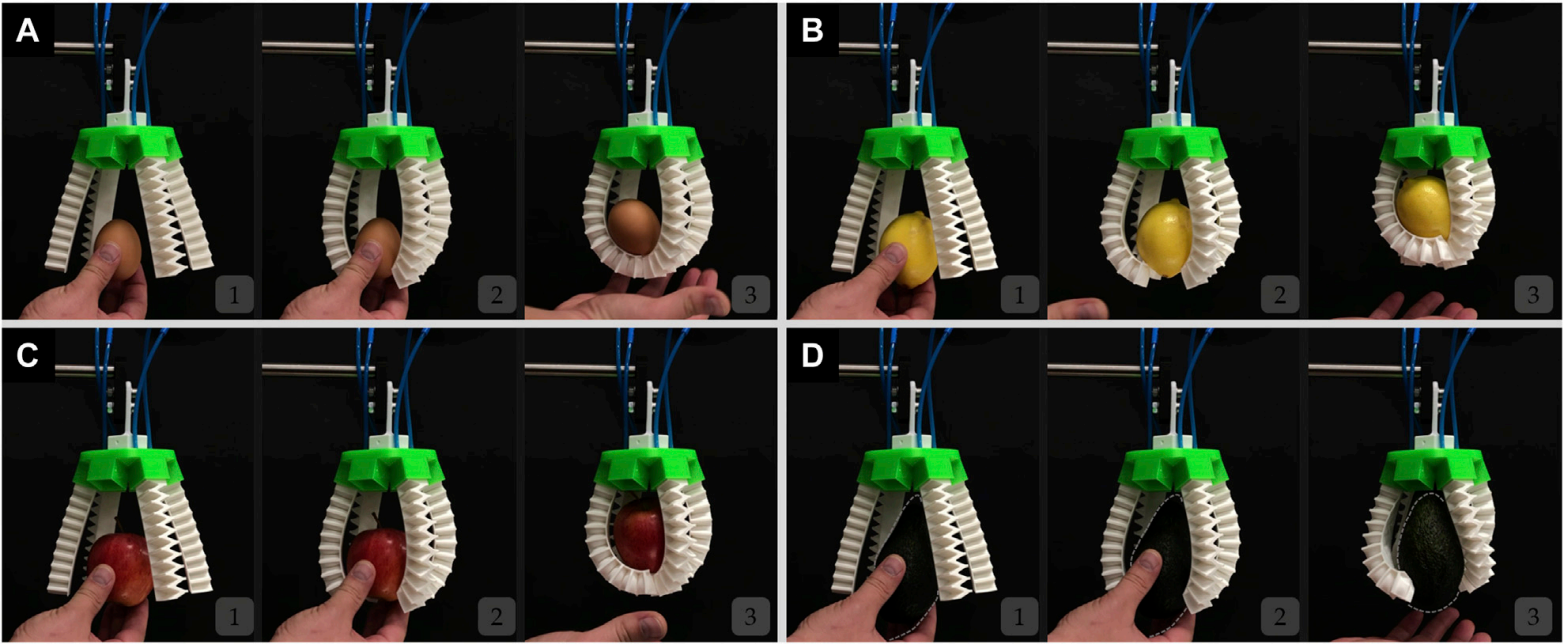
The soft modular gripper in a four-finger configuration without the mechanical metamaterial attempts to but fails to grasp **(A)** an egg, **(B)** a lemon, **(C)** an apple, **(D)** and fails to grasp stably an avocado.

Similarly, the four-finger configuration modular gripper without the inclusion of the mechanical metamaterial was not capable of grasping the egg, lemon, and apple as shown in Figure 11 and Supplementary Video S4. The egg, lemon, and apple were pushed upward by the curling motion of the fingers and their out-of-plane deformation leading to unsuccessful grasps where the objects laid only on the closed finger and became stuck between the fingers and the rigid base. For the avocado, although the soft gripper could grasp it, again, the fingers curled leading to out-of-plane deformations which in turn led to an unstable grip (Figure 11 and Supplementary Video S4).

**FIGURE 12:**
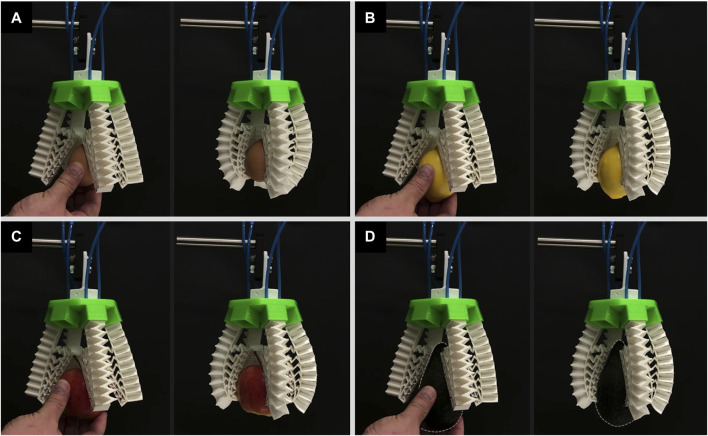
The soft modular gripper in a four-finger configuration with the mechanical metamaterial grasping successfully **(A)** an egg, **(B)** a lemon, **(C)** an apple, and **(D)** an avocado.

Moreover, the same four-finger gripper with an integrated mechanical metamaterial was able to successfully grasp all the objects by conforming to their shape and it has alleviated the out-of-plane deformation behavior encountered in this configuration as well and thus leading to a stable and firm grasp as shown in Figure 12 and Supplementary Video S4. Although the fingers may exhibit a curling behavior due to unequal grasping angles or forces, the mechanical metamaterial ensures effective and successful grasping by compensating for such imperfections with higher conformability and reduced out-of-plane deformations.

## 4 Discussion

It is important to note that the absence of the metamaterial is not solely the main reason for failed grasps. Other important factors that contribute to a failed grasp include spacing between the fingers, length of the fingers, pre-grasp pose, the orientation of the object, and the bending behavior (i.e., curvature) of the soft fingers. However, in this work, all these parameters were held constant, and it was proven that for such constant parameters the inclusion of the metamaterial lead to successful grasps.

Also, it is important to note that although the metamaterial enhanced the conformability of the gripper and reduced the out-of-plane deformation of the fingers it did not provide a highly soft structure that can perfectly take the shape of the object in contact (i.e., highly deformable structure) (Figure 6B). This is mainly due to the property of the TPU used. Although the TPU is soft it not as soft as silicone and still has some moderate degree of resistance to deformation. In addition, the surface of the printed metamaterials is shiny and smooth which in some cases reduces the contact friction that enhances the stability of the grasps. In future work, these limitations will be addressed by changing the choice of the material for the metamaterial by testing the behavior of different materials and their surface finish properties.

## 5 Conclusion

We have developed a 3D printed modular soft pneumatic gripper integrated with mechanical metamaterials for conformal grasping. The active component of the gripper consists of soft monolithic pneumatic fingers that generate a bending motion upon actuation while the passive component consists of a mechanical metamaterial that consists of an auxetic structure and compliant ribs for enhancing the conformability of the soft gripper. This design proved its significance for versatile soft modular grippers, and the importance of design along with material properties.

The soft gripper could successfully grasp different objects with the inclusion of the mechanical metamaterial in three different configurations including two, three, and four-finger configurations. The addition of the mechanical metamaterial proved not only that the gripper succeeds in grasping the objects or stably grasping them, but it also proved that the added capability of conformability leads to reduced out-of-plane deformations that also increased the gripping stability and consequently enhanced the grasping performance of the soft modular gripper. Future studies will include testing the soft gripper in a dynamic scheme by attaching the soft modular gripper to an industrial robotic manipulator to pick and place a wide variety of objects under different dynamic conditions.

## Data Availability

The raw data supporting the conclusion of this article will be made available by the authors, without undue reservation.

## References

[B1] Abondance S. Teeple C. B. Wood R. J. (2020). A Dexterous Soft Robotic Hand for Delicate In-Hand Manipulation. IEEE Robot. Autom. Lett. 5 (4), 5502–5509. 10.1109/LRA.2020.3007411

[B2] Alici G. Canty T. Mutlu R. Hu W. Sencadas V. (2018). Modeling and Experimental Evaluation of Bending Behavior of Soft Pneumatic Actuators Made of Discrete Actuation Chambers. Soft Robotics 5 (1), 24–35. 10.1089/soro.2016.0052 29412079

[B3] Alici G. (2018). Softer Is Harder: What Differentiates Soft Robotics from Hard Robotics? MRS Adv. 3 (28), 1557–1568. 10.1557/adv.2018.159

[B4] Calisti M. Picardi G. Laschi C. (2017). Fundamentals of Soft Robot Locomotion. J. R. Soc. Interf. 14 (130), 20170101. 10.1098/rsif.2017.0101 PMC5454300 28539483

[B5] Chen F. Xu W. Zhang H. Wang Y. Cao J. Wang M. Y. (2018). Topology Optimized Design, Fabrication, and Characterization of a Soft Cable-Driven Gripper. IEEE Robot. Autom. Lett. 3 (3), 2463–2470. 10.1109/LRA.2018.2800115

[B6] Cianchetti M. Laschi C. Menciassi A. Dario P. (2018). Biomedical Applications of Soft Robotics. Nat. Rev. Mater. 3 (6), 143–153. 10.1038/s41578-018-0022-y

[B7] Deimel R. Brock O. (2016). A Novel Type of Compliant and Underactuated Robotic Hand for Dexterous Grasping. Int. J. Robotics Res. 35 (1-3), 161–185. 10.1177/0278364915592961

[B8] Fatahillah M. Oh N. Rodrigue H. (2020). A Novel Soft Bending Actuator Using Combined Positive and Negative Pressures. Front. Bioeng. Biotechnol. 8, 472. 10.3389/fbioe.2020.00472 32509752 PMC7248170

[B9] Fraś J. Maciaś M. Czubaczyński F. Sałek P. Główka J. (2017). “Soft Flexible Gripper Design, Characterization and Application,” in Recent Advances in Systems, Control and Information Technology. Editors Szewczyk R. Kaliczyńska M. (Poland: Springer International Publishing), 368–377.

[B10] Galloway K. C. Polygerinos P. Walsh C. J. Wood R. J. (2013). “Mechanically Programmable bend Radius for Fiber-Reinforced Soft Actuators,” in 2013 16th International Conference on Advanced Robotics (ICAR), Montevideo, Uruguay, 25-29 Nov. 2013 (IEEE), 1–6. 10.1109/icar.2013.6766586

[B11] Glick P. Suresh S. A. Ruffatto D. Cutkosky M. Tolley M. T. Parness A. (2018). A Soft Robotic Gripper with Gecko-Inspired Adhesive. IEEE Robot. Autom. Lett. 3 (2), 903–910. 10.1109/LRA.2018.2792688

[B12] Hao Y. Biswas S. Hawkes E. W. Wang T. Zhu M. Wen L. (2021). A Multimodal, Enveloping Soft Gripper: Shape Conformation, Bioinspired Adhesion, and Expansion-Driven Suction. IEEE Trans. Robot. 37 (2), 350–362. 10.1109/TRO.2020.3021427

[B13] Hao Y. Gong Z. Xie Z. Guan S. Yang X. Ren Z. (2016). Universal Soft Pneumatic Robotic Gripper with Variable Effective Length, in 2016 35th Chinese Control Conference CCC, Chengdu, China, 27-29 July 2016 (IEEE), 6109–6114. 10.1109/chicc.2016.7554316

[B14] Hu W. Alici G. (2020). Bioinspired Three-Dimensional-Printed Helical Soft Pneumatic Actuators and Their Characterization. Soft Robotics 7 (3), 267–282. 10.1089/soro.2019.0015 31687877

[B15] Hussain I. Al-Ketan O. Renda F. Malvezzi M. Prattichizzo D. Seneviratne L. (2020). Design and Prototyping Soft-Rigid Tendon-Driven Modular Grippers Using Interpenetrating Phase Composites Materials. Int. J. Robotics Res. 39 (14), 1635–1646. 10.1177/0278364920907697

[B16] Kaur M. Kim W. S. (2019). Toward a Smart Compliant Robotic Gripper Equipped with 3D‐Designed Cellular Fingers. Adv. Intell. Syst. 1 (3), 1900019. 10.1002/aisy.201900019

[B17] Ke X. Jang J. Chai Z. Yong H. Zhu J. Chen H. (2021). Stiffness Preprogrammable Soft Bending Pneumatic Actuators for High-Efficient, Conformal Operation. Soft Robotics. 10.1089/soro.2020.0207 34255577

[B18] Keong B. A. W. Hua R. Y. C. (2018). A Novel Fold-Based Design Approach toward Printable Soft Robotics Using Flexible 3D Printing Materials. Adv. Mater. Technol. 3 (2), 1700172. 10.1002/admt.201700172

[B19] Koivikko A. Drotlef D.-M. Dayan C. B. Sariola V. Sitti M. (2021). 3D‐Printed Pneumatically Controlled Soft Suction Cups for Gripping Fragile, Small, and Rough Objects. Adv. Intell. Syst. 3 (9), 2100034. 10.1002/aisy.202100034

[B20] Laschi C. Cianchetti M. (2014). Soft Robotics: New Perspectives for Robot Bodyware and Control. Front. Bioeng. Biotechnol. 2, 3. 10.3389/fbioe.2014.00003 25022259 PMC4090912

[B21] Li S. Stampfli J. J. Xu H. J. Malkin E. Diaz E. V. Rus D. (2019). “A Vacuum-Driven Origami “Magic-ball” Soft Gripper,” in 2019 International Conference on Robotics and Automation (ICRA), Montreal Convention Centre, 20 May 2019–24 May 2019 (IEEE), 7401–7408.

[B22] Lin H. T. Leisk G. G. Trimmer B. (2011). GoQBot: A Caterpillar-Inspired Soft-Bodied Rolling Robot. Bioinspir. Biomim. 6 (2), 026007. 10.1088/1748-3182/6/2/026007 21521905

[B23] MacCurdy R. Katzschmann R. Youbin K. Rus D. (2016). “Printable Hydraulics: A Method for Fabricating Robots by 3D Co-printing Solids and Liquids,” in IEEE International Conference on Robotics and Automation (ICRA), Stockholm, Sweden, 16-21 May 2016 (IEEE), 3878–3885.

[B24] Marchese A. D. Katzschmann R. K. Rus D. (2015). A Recipe for Soft Fluidic Elastomer Robots. Soft Robotics 2 (1), 7–25. 10.1089/soro.2014.0022 27625913 PMC4997626

[B25] Mosadegh B. Polygerinos P. Keplinger C. Wennstedt S. Shepherd R. F. Gupta U. (2014). Pneumatic Networks for Soft Robotics that Actuate Rapidly. Adv. Funct. Mater. 24 (15), 2163–2170. 10.1002/adfm.201303288

[B26] Mutlu R. Alici G. in het Panhuis M. Spinks G. M. (2016). 3D Printed Flexure Hinges for Soft Monolithic Prosthetic Fingers. Soft Robotics 3 (3), 120–133. 10.1089/soro.2016.0026

[B27] Mutlu R. Tawk C. Alici G. Sariyildiz E. (2017). “A 3D Printed Monolithic Soft Gripper with Adjustable Stiffness,” in IECON 2017 - 43rd Annual Conference of the IEEE Industrial Electronics Society, Beijing, China, 29 Oct.-1 Nov. 2017 (IEEE), 6235–6240. 10.1109/iecon.2017.8217084

[B28] Pagoli A. Chapelle F. Corrales J. A. Mezouar Y. Lapusta Y. (2021). A Soft Robotic Gripper with an Active Palm and Reconfigurable Fingers for Fully Dexterous In-Hand Manipulation *. IEEE Robot. Autom. Lett. 6, 7706–7713. 10.1109/LRA.2021.3098803

[B29] Pham D. T. Yeo S. H. (1991). Strategies for Gripper Design and Selection in Robotic Assembly. Int. J. Prod. Res. 29 (2), 303–316. 10.1080/00207549108930072

[B30] Schaffner M. Faber J. A. Pianegonda L. Rühs P. A. Coulter F. Studart A. R. (2018). 3D Printing of Robotic Soft Actuators with Programmable Bioinspired Architectures. Nat. Commun. 9 (1), 878. 10.1038/s41467-018-03216-w 29491371 PMC5830454

[B31] Scharff R. B. N. Wu J. Geraedts J. M. P. Wang C. C. L. (2019). “Reducing Out-Of-Plane Deformation of Soft Robotic Actuators for Stable Grasping,” in 2019 2nd IEEE International Conference on Soft Robotics (RoboSoft), Seoul, Korea, 14 Apr 2019-18 Apr 2019 (IEEE), 265–270.

[B32] Shintake J. Cacucciolo V. Floreano D. Shea H. (2018). Soft Robotic Grippers. Adv. Mater. 30 (29), 1707035. 10.1002/adma.201707035 29736928

[B33] Sui D. Zhu Y. Zhao S. Wang T. Agrawal S. K. Zhang H. (2020). A Bioinspired Soft Swallowing Gripper for Universal Adaptable Grasping. Soft Robotics. 10.1089/soro.2019.0106 33275516

[B34] Tawk C. Alici G. (2021). A Review of 3D‐Printable Soft Pneumatic Actuators and Sensors: Research Challenges and Opportunities. Adv. Intell. Syst. 3 (6), 2000223. 10.1002/aisy.202000223

[B35] Tawk C. Alici G. (2020). Finite Element Modeling in the Design Process of 3D Printed Pneumatic Soft Actuators and Sensors. Robotics 9, 52. 10.3390/robotics9030052

[B36] Tawk C. Gao Y. Mutlu R. Alici G. (2019a). “Fully 3D Printed Monolithic Soft Gripper with High Conformal Grasping Capability,” in 2019 IEEE/ASME International Conference on Advanced Intelligent Mechatronics (AIM), Hong Kong, China, 8-12 July 2019 (IEEE), 1139–1144.

[B37] Tawk C. Gillett A. in het Panhuis M. Spinks G. M. Alici G. (2019b). A 3D-Printed Omni-Purpose Soft Gripper. IEEE Trans. Robot. 35 (5), 1268–1275. 10.1109/tro.2019.2924386

[B38] Tawk C. in het Panhuis M. Spinks G. M. Alici G. (2018). Bioinspired 3D Printable Soft Vacuum Actuators for Locomotion Robots, Grippers and Artificial Muscles. Soft Robotics 5 (6), 685–694. 10.1089/soro.2018.0021 30040042

[B39] Tawk C. Mutlu R. Alici G. (2020). “A3D Printed Modular Soft Gripper for Conformal Grasping,” in 2020 IEEE/ASME International Conference on Advanced Intelligent Mechatronics (AIM), Boston, MA, USA, 6-9 July 2020 (IEEE), 583–588. 10.1109/aim43001.2020.9158818

[B40] Tawk C. Spinks G. M. in het Panhuis M. Alici G. (2019c). 3D Printable Linear Soft Vacuum Actuators: Their Modeling, Performance Quantification and Application in Soft Robotic Systems. Ieee/asme Trans. Mechatron. 24 (5), 2118–2129. 10.1109/TMECH.2019.2933027

[B41] Trivedi D. Rahn C. D. Kier W. M. Walker I. D. (2008). Soft Robotics: Biological Inspiration, State of the Art, and Future Research. Appl. Bionics Biomech. 5 (3), 99–117. 10.1080/11762320802557865

[B42] Wang T. Ge L. Gu G. (2018). Programmable Design of Soft Pneu-Net Actuators with Oblique chambers Can Generate Coupled Bending and Twisting Motions. Sensors Actuators A: Phys. 271, 131–138. 10.1016/j.sna.2018.01.018

[B43] Whitesides G. M. (2018). Soft Robotics. Angew. Chem. Int. Ed. 57 (16), 4258–4273. 10.1002/anie.201800907 29517838

[B44] Xavier M. S. Fleming A. J. Yong Y. K. (2021). Finite Element Modeling of Soft Fluidic Actuators: Overview and Recent Developments. Adv. Intell. Syst. 3 (2), 2000187. 10.1002/aisy.202000187

[B45] Yang Y. Vella K. Holmes D. P. (2021). Grasping with Kirigami Shells. Sci. Robot. 6 (54), eabd6426. 10.1126/scirobotics.abd6426 34043535

[B46] Yap H. K. Ng H. Y. Yeow C.-H. (2016). High-Force Soft Printable Pneumatics for Soft Robotic Applications. Soft Robotics 3 (3), 144–158. 10.1089/soro.2016.0030

[B47] Yirmibeşoğlu O. D. Oshiro T. Olson G. Palmer C. Mengüç Y. (2019). Evaluation of 3D Printed Soft Robots in Radiation Environments and Comparison with Molded Counterparts. Front. Robot. AI 6, 40. 10.3389/frobt.2019.00040 33501056 PMC7805716

[B48] Zang H. Liao B. Lang X. Zhao Z.-L. Yuan W. Feng X.-Q. (2020). Bionic Torus as a Self-Adaptive Soft Grasper in Robots. Appl. Phys. Lett. 116, 023701. 10.1063/1.5128474

[B49] Zhang H. Kumar A. S. Fuh J. Y. H. Wang M. Y. (2018). “Topology Optimized Design, Fabrication and Evaluation of a Multimaterial Soft Gripper,” in 2018 IEEE International Conference on Soft Robotics (RoboSoft), Livorno, Italy, 24-28 April 2018 (IEEE), 424–430.

[B50] Zhou J. Chen S. Wang Z. (2017). A Soft-Robotic Gripper with Enhanced Object Adaptation and Grasping Reliability. IEEE Robot. Autom. Lett. 2 (4), 2287–2293. 10.1109/LRA.2017.2716445

[B51] Zhou L. Ren L. Chen Y. Niu S. Han Z. Ren L. (2021). Bio‐Inspired Soft Grippers Based on Impactive Gripping. Adv. Sci. 8 (9), 2002017. 10.1002/advs.202002017 PMC8097330 33977041

[B52] Zhou X. Majidi C. O’Reilly O. M. (2015). Soft Hands: An Analysis of Some Gripping Mechanisms in Soft Robot Design. Int. J. Sol. Structures 64-65, 155–165. 10.1016/j.ijsolstr.2015.03.021

[B53] Zolfagharian A. Gharaie S. Gregory J. Bodaghi M. Kaynak A. Nahavandi S. (2021). A Bioinspired Compliant 3D-Printed Soft Gripper. Soft Robotics. 10.1089/soro.2020.0194 34297904

